# Time-resolved simulation of blood flow through left anterior descending coronary artery: effect of varying extent of stenosis on hemodynamics

**DOI:** 10.1186/s12872-023-03190-2

**Published:** 2023-03-27

**Authors:** Yinghong Zhao, Huihui Wang, Wenbing Chen, Wenyue Sun, Xianchao Yu, Cunjie Sun, Gang Hua

**Affiliations:** 1 China University of Mining and Technology, No.1, Daxue Road, Xuzhou, Jiangsu China; 2 Xuzhou Medical University, 209 Tongshan Road, Xuzhou, Jiangsu China; 3 Shenzhen University, Shenzhen, Guangdong China; 4 School of Life Science and Technology, University of Electronic Science and Technology of China, Chengdu, China; 5 The Affiliated Hospital of Xuzhou Medical University, Xuzhou, Jiangsu China

**Keywords:** Left anterior descending coronary arteries, Stenosis, Hemodynamics, CFD, Simulation

## Abstract

**Background and objectives:**

Real-time blood flow variation is crucial for understanding the dynamic development of coronary atherosclerosis. The main objective of this study is to investigate the effect of varying extent of stenosis on the hemodynamic features in left anterior descending coronary artery.

**Methods:**

Various Computational fluid dynamics (CFD) models were constructed with patient-specific CT image data, using actual fractional flow reserve (FFR) as boundary conditions to provide a real-time quantitative description of hemodynamic properties. The hemodynamic parameters, such as the local and instantaneous wall shear stress (WSS), oscillating shear index (OSI) and relative residence time (RRT), blood flow velocity and pressure drop during various phases of cardiac cycle were provided in detail.

**Results:**

There was no evident variation in hemodynamic parameters in the cases of less than 50% stenosis while there were abrupt and dramatic changes in hemodynamics when the stenosis aggravated from 60 to 70%. Furthermore, when the stenosis was beyond 70%, there existed substantial pressure difference, WSS, and blood flow velocity in the center of the stenosis. Although OSI and RRT increased along with the aggravation of stenosis, they appeared with obvious abnormalities across all cases, even in mild stenosis.

**Conclusion:**

The simulation could present a dynamic and comprehensive profile of how hemodynamic parameters vary in accordance with divergent severities of stenosis, which could serve as an effective reference for the clinicians to have a deeper insight into the pathological mechanism of coronary atherosclerosis and stenosis.

## Introduction

Coronary heart disease (CHD) is a cardiovascular disease in which atherosclerotic lesions occur in coronary arteries, resulting in vascular stenosis or obstruction. In severe cases, it will cause myocardial ischemia, hypoxia or necrosis [1]. Therefore, prevention of CHD further episodes and inhibition on the progression of coronary atherosclerosis are pivotal [2]. Consequently, it is warranted to observe the surge of research into the pathogenesis of coronary stenosis lesion in the literature [3–6], which could enable clinicians to make more informed decisions when performing medical interventions. Among these studies, hemodynamic numerical simulation by means of computational fluid dynamics (CFD) simulation is deemed as a well-established method to study the hemodynamic mechanism of cardiovascular diseases [7–9], on the ground that it could quantify and three-dimensionally display hemodynamic distribution in coronary stenosis, thus providing a better understanding of the physiological and pathological basis of the lesion.

Currently, most studies mainly focus on the destruction and changes of endothelial cells in coronary stenotic vessels due to physical factors under different flow fields and action time [10–15], which could provide a rich theoretical basis for exploring the pathological mechanism of atherosclerosis formation and its development. Moreover, current research reveals that coronary atherosclerosis is also the process of the local narrowing of coronary arteries, and that its stenosis lesion enables the significant changes of the geometry and physiological properties of coronary arteries, the cause of the alteration in blood flow pattern and flow field simultaneously, and the induction of the proliferation of endothelial tissue, which would promote further stenosis of coronary arteries and the aggravation of atherosclerosis, thus leading to serious circulatory diseases [15–21].

However, the present studies tend to focus more on comparatively severe stenosis (≥ 70%), especially the stenosis which is over 80%, without paying adequate attention to investigating the dynamic progression of hemodynamic distribution in relatively moderate and mild stenosis (< 70%) [8, 20–23]. Although comparatively less severe, moderate and mild stenosis still deserve due attention and thorough investigation by the researchers due to the fact that they have the potential to pose health hazards for the patients as well. For example, studies have shown that coronary stenosis ranging from 50 to 70% may constitute the mechanism of most chronic stable angina attacks [20–22]. Furthermore, to date, there is a paucity of studies on investigating how hemodynamic parameters vary in line with gradual aggravation of stenosis of coronary arteries across relatively large varying extent of stenosis from mild to severe degrees. Such studies have the merits of rendering clinicians in a more favorable position to have a panoramic and dynamic view of how the coronary atherosclerosis progresses and develops, as well as keeping clinicians well-informed about from what extent of stenosis they should pay heed to the condition before the further deterioration of the disease with too much detrimental consequence based on the hemodynamic evidence. Additionally, in current studies, either an ideal physical vessel model or non-patient specific boundary conditions are frequently adopted, thus failing to elucidate the detailed and more accurate hemodynamic mechanism, which highlights the needs for further studies on time-resolution and quantitative numerical simulation of coronary hemodynamics based on individualized clinical data [8, 22–25]. Therefore, this study intends to establish patient-specific left anterior descending (LAD) coronary artery models with a wide range of stenosis from relatively mild to severe degrees to simulate the reliable pulsatile flow status and calculate the multi-dimensional hemodynamic parameters. Moreover, this study also aims to provide a real-time quantitative description of hemodynamic properties by adopting actual fractional flow reserve (FFR) as initial setting to monitor patient-dependent pressure [8]. In addition, this study would conduct a quantitative analysis of the correlation between the extent of coronary artery stenosis and the time-resolved hemodynamic changes as well. Accordingly, the results of this study could serve as an effective reference for the clinicians to have a deeper insight into the pathological mechanism of coronary atherosclerosis.

## Materials and methods

### Coronary vascular modelling

**Fig. 1:**
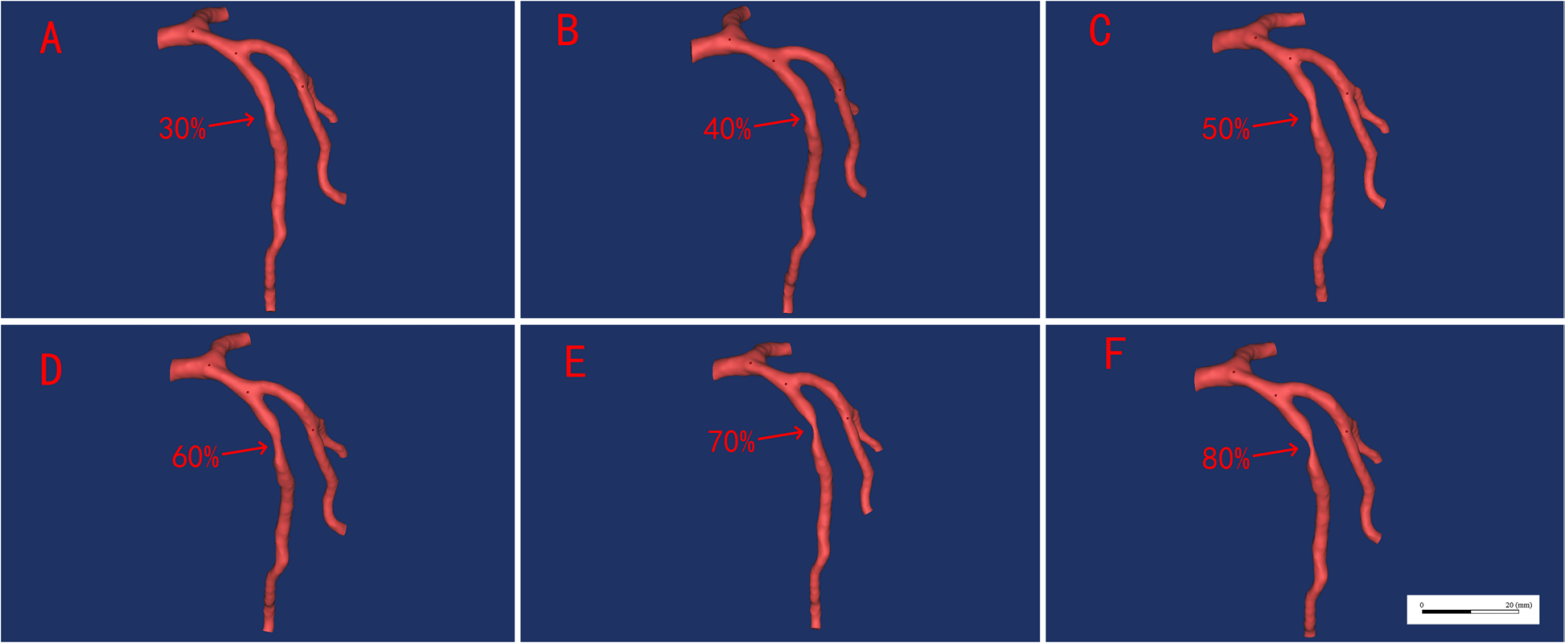
Demonstration of multi-stenosis LAD coronary artery models

In this study, 6 cases with various degrees of stenosis in LAD (30%-80% stenosis) were selected and all subjects were performed on a 256-multilayer CT scanner with slices thickness of 0.625 mm. The CT image data was imported into Mimics 21.0 (Materialise, Leuven, Belgium), a medical image processing software, with DICOM (digital imaging and communications in medicine) format for data processing and three-dimensional (3D) reconstruction, to obtain geometric models of the patient's LAD coronary artery. The subsequent segmentation was performed with multi-threshold adaptive algorithm. The initial gray level characteristics of LADs were evaluated and corrected by technicians. The LADs were separated from other tissues after segmentation. The 3D reconstruction models of the LAD were created after smooth processing while retaining the original physiological and anatomical properties, as illustrated in Fig. 1.

### Governing equations and boundary conditions

Blood is assumed to be homogenous and non-Newtonian fluid in this computational fluid dynamics (CFD) simulation. The following are the governing equations for blood flow [26, 27]:

1 $$\rho \left(\frac{\partial u}{\partial t}+u\bullet \nabla u\right)=-\nabla p+\nabla \bullet \tau$$

2 $$\nabla \bullet u=0$$

where $u$ represents the velocities, $t$ is the time, $\rho$ is the fluid density at 1,060 kg/m^3^, and $p$ is the pressure, $\tau$ represents the stress tensor.

3 $$\tau =2\eta \left(\dot{\gamma }\right)D$$

$D$ and $\dot{\gamma }$ represents deformation tensor rate and shear rate, $\eta$ is the blood viscosity. Carreau model was used to calculate the blood viscosity [26].

4 $$\eta \left(\dot{\gamma }\right)={\eta }_{\infty }+\left({\eta }_{0}-{\eta }_{\infty }\right){\left[1+{\left(\lambda \dot{\gamma }\right)}^{2}\right]}^{\frac{n-1}{2}}$$

All values were taken from the literature, $\eta_\infty(\mathrm{high}\;\mathrm{shear}\;\mathrm{viscosity})=3.45\times10^{-3}Kg/(m\bullet s)$, $\eta_0(\mathrm{low}\;\mathrm{shear}\;\mathrm{viscosity})=5.6\times10^{-2}Kg/(m\bullet s)$, $n(\mathrm{power}\;\mathrm{law}\;\mathrm{index})=0.3568$, $\lambda(\mathrm{time}\;\mathrm{constant})=3.313s$ [26].

**Fig. 2:**
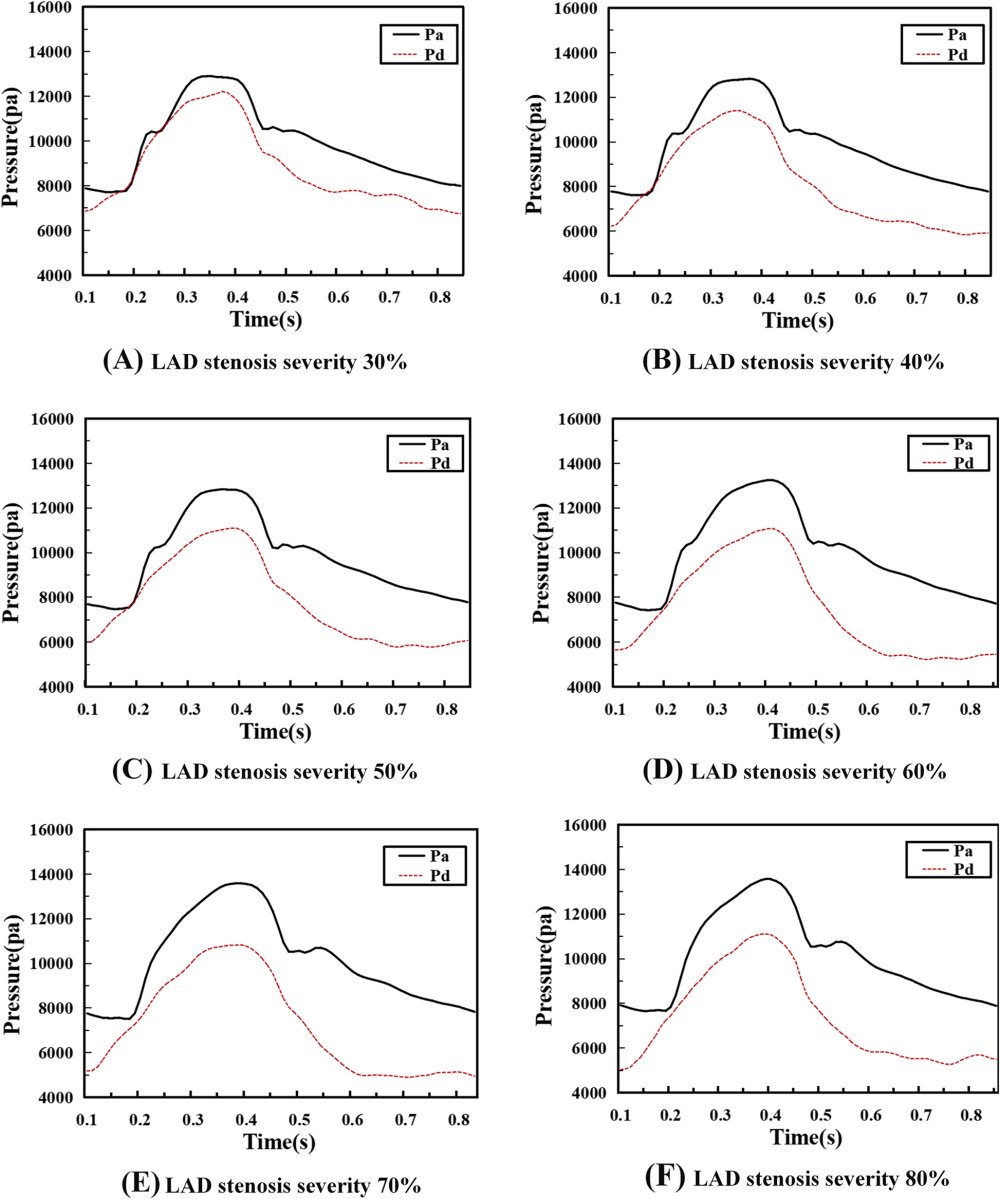
Fitted curves of blood pressure proximal to the stenosis Pa(t) and the pressure distal to the stenosis Pd(t) used for inlet flow boundary condition based on invasive FFR measurements in vivo

The 3D models of the patient-specific LAD were imported into ANSYS Fluent 2020 R1 (Ansys Inc., Canonsburg, PA, USA) for mesh generation. Fine tetrahedral mesh was established, and the number of grids and its quality was shown in Table 1. Mesh independence tests indicated that above 1.5 million cells, the results did not change significantly (∼2%) for outlet velocities. The pressure values (Fig. 2) fitted from the clinical measured FFR data of patients were used to define the inlet boundary conditions for LAD models. FFR is the ratio of maximal blood flow distal to a stenotic lesion to maximal flow in the same artery if hypothetically normal [9]. Pressure flow waveforms in the LADs were obtained from the subjects with FFR reflecting the physiologically pulsatile, biphasic blood flow from the coronary arteries into the LAD. The average of the pressures distal to the stenosis measured was applied to the setting of the outlet. For the models (from 30% stenosis to 80% stenosis), the outlets were set respectively as 8843Pa, 8041Pa, 7819Pa, 7446Pa, 7377Pa, and 7229Pa.

**Table 1:** Grid numbers in the reconstructed LAD models

Stenosis severity	Grids	Cells	Grid quality^a^
30%	365,664	1,889,408	0.83571
40%	391,906	2,025,915	0.83565
50%	395,853	2,049,530	0.83598
60%	421,738	2,182,703	0.83589
70%	427,895	2,215,701	0.83572
80%	426,284	2,206,591	0.83583

^a^The content of grid quality value is between 0 and 1; 0 is the worst and 1 is the best. Generally, this value is greater than 0.7, indicating that the model can be used for simulation calculation. The mesh quality of the models established in this experiment is greater than 0.83, and its qualities could ensure the validity of the simulation results

Since the blood flow in the coronary artery is a turbulent flow, current study used shear stress turbulence model to account for turbulent velocity due to its accuracy in predicting the turbulent behavior of blood in coronary arteries [9, 26, 27]. Additionally, the residual convergence criteria were set as 10^−4^.

Hemodynamic flow velocity, pressure, wall shear stress (WSS), oscillatory shear index (OSI) and relative residence time (RRT) were calculated. It is well acknowledged by the literature that the activation of endothelial cell by the shear stress and localization of plaque are closely related [27]. The WSS is calculated based on the following equation.

5 $$WSS=\frac{1}{T}\underset{0}{\overset{T}{\int }}\left|\frac{\mu \partial {v}_{t}}{\partial n}\right|dt$$

where $\mu$ is blood viscosity, ${v}_{t}$ is the velocity vector near wall perpendicular to the surface, $n$ is distance to the wall surface and $T$ is pulsatile period dt is the time derivative of the local shear stress [27].

OSI is an index frequently used to evaluate the change of axial direction in WSS within the cardiac cycle. Abnormal OSI often indicates the drastic disturbance of flow field, which is supposed to be associated with the formation of thrombosis [28]. OSI is defined as follows:

6 $$\mathrm{OSI}=0.5\times \left[1-\frac{\left|{\int }_{0}^{T}WSSdt\right|}{{\int }_{0}^{T}\left|WSS\right|dt}\right]$$

where T is the period of the cardiac cycle, and WSS is the WSS vector.

Relative residence time (RRT) gives a relative estimation of the residence time of the fluid in an area. Its value increases in the areas where the near-wall velocity has large direction changes but small magnitude over one period [29]. RRT is computed according to the following formulas:

7 $$\mathrm{RRT}={\left[\left(1-2\times \mathrm{OSI}\right)\times \mathrm{TAWSS}\right]}^{-1}$$

8 $$\mathrm{TAWSS}=\frac{1}{\mathrm{T}}{\int }_{0}^{T}\left|WSS\right|dt$$

where T is the period of the cardiac cycle, and WSS is the WSS vector.

## Results

**Fig. 3:**
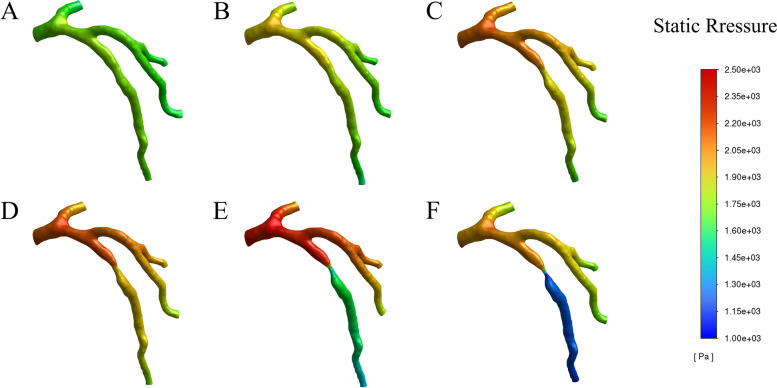
Variation of pressure distribution at the peak systole in the cardiac cycle. **A**. 30% stenosis, **B**. 40% stenosis, **C**. 50% stenosis, **D**. 60% stenosis, **E**. 70% stenosis, **F**. 80% stenosis

**Fig. 4:**
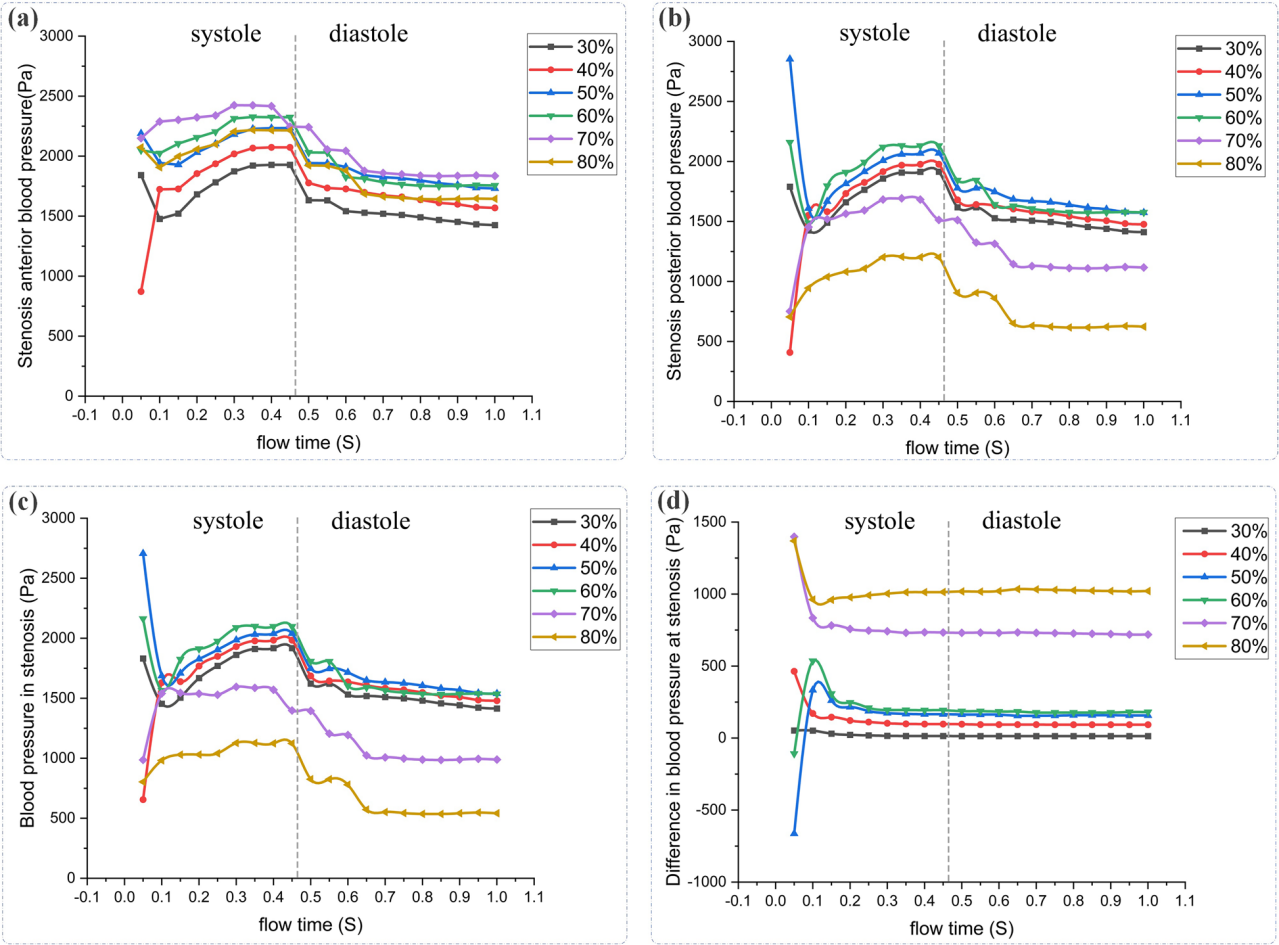
Variation of pressure distribution at specific points (**A**, anterior stenosis; **B**, posterior stenosis; **C**, in stenosis (the center); **D**, pressure difference in the center) during the cycle

**Fig. 5:**
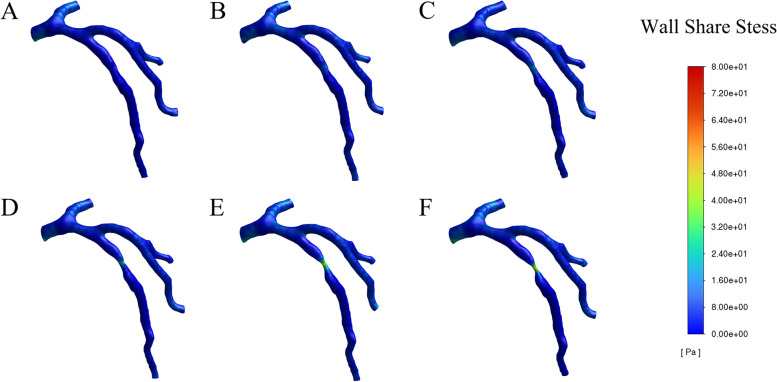
Wall shear stress (WSS) distribution in the LAD at the peak systole in a cardiac cycle. **A**. 30% stenosis, **B**. 40% stenosis, **C**. 50% stenosis, **D**. 60% stenosis, **E**. 70% stenosis, **F**. 80% stenosis

**Fig. 6:**
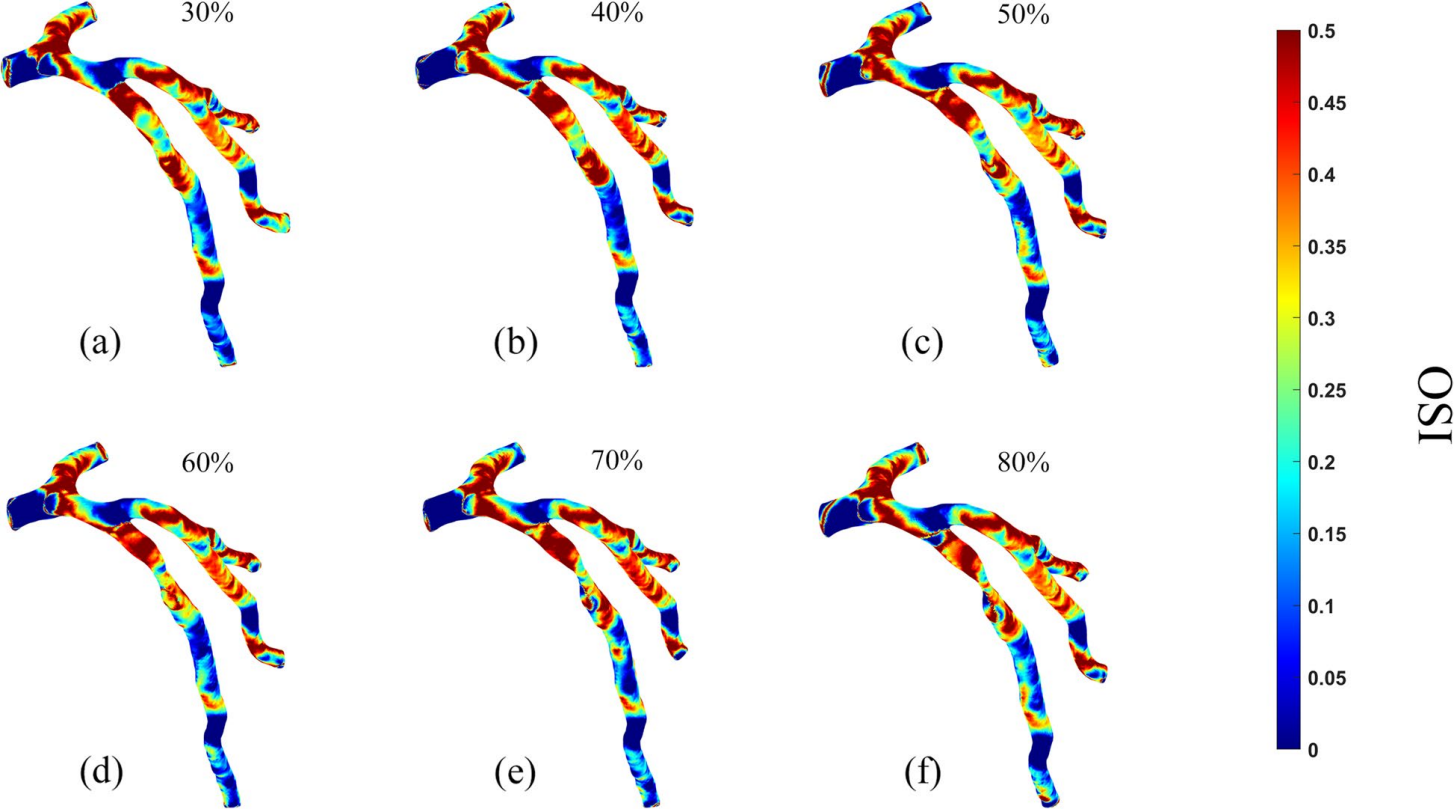
Oscillatory Shear Index (OSI) distribution on the vessel walls in six patients. **A**. 30% stenosis, **B**. 40% stenosis, **C**. 50% stenosis, **D**. 60% stenosis, **E**. 70% stenosis, **F**. 80% stenosis

**Fig. 7:**
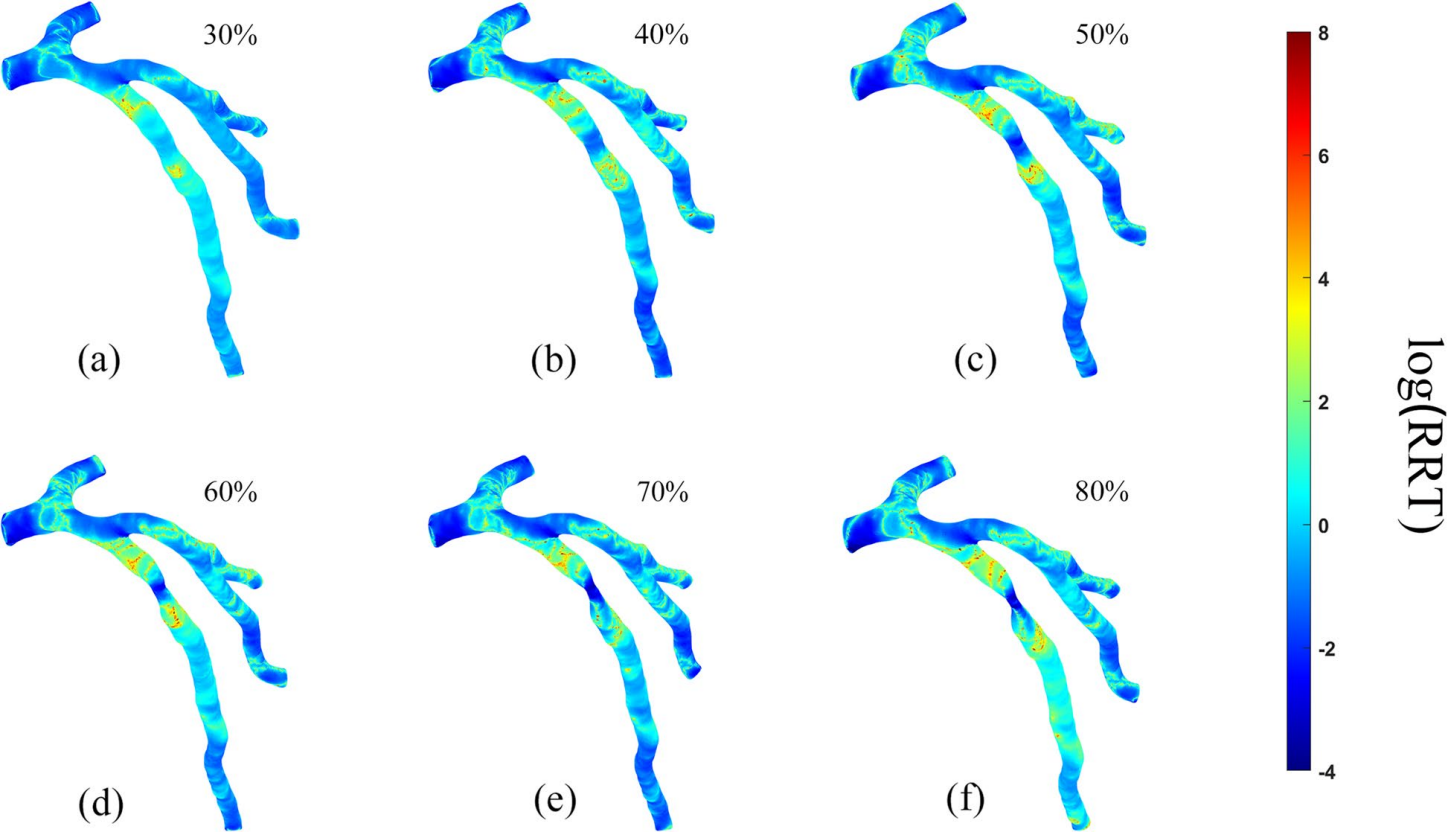
Relative residence time (RRT) distribution on the vessel walls in six patients. **A**. 30% stenosis, **B**. 40% stenosis, **C**. 50% stenosis, **D**. 60% stenosis, **E**. 70% stenosis, **F**. 80% stenosis

**Fig. 8:**
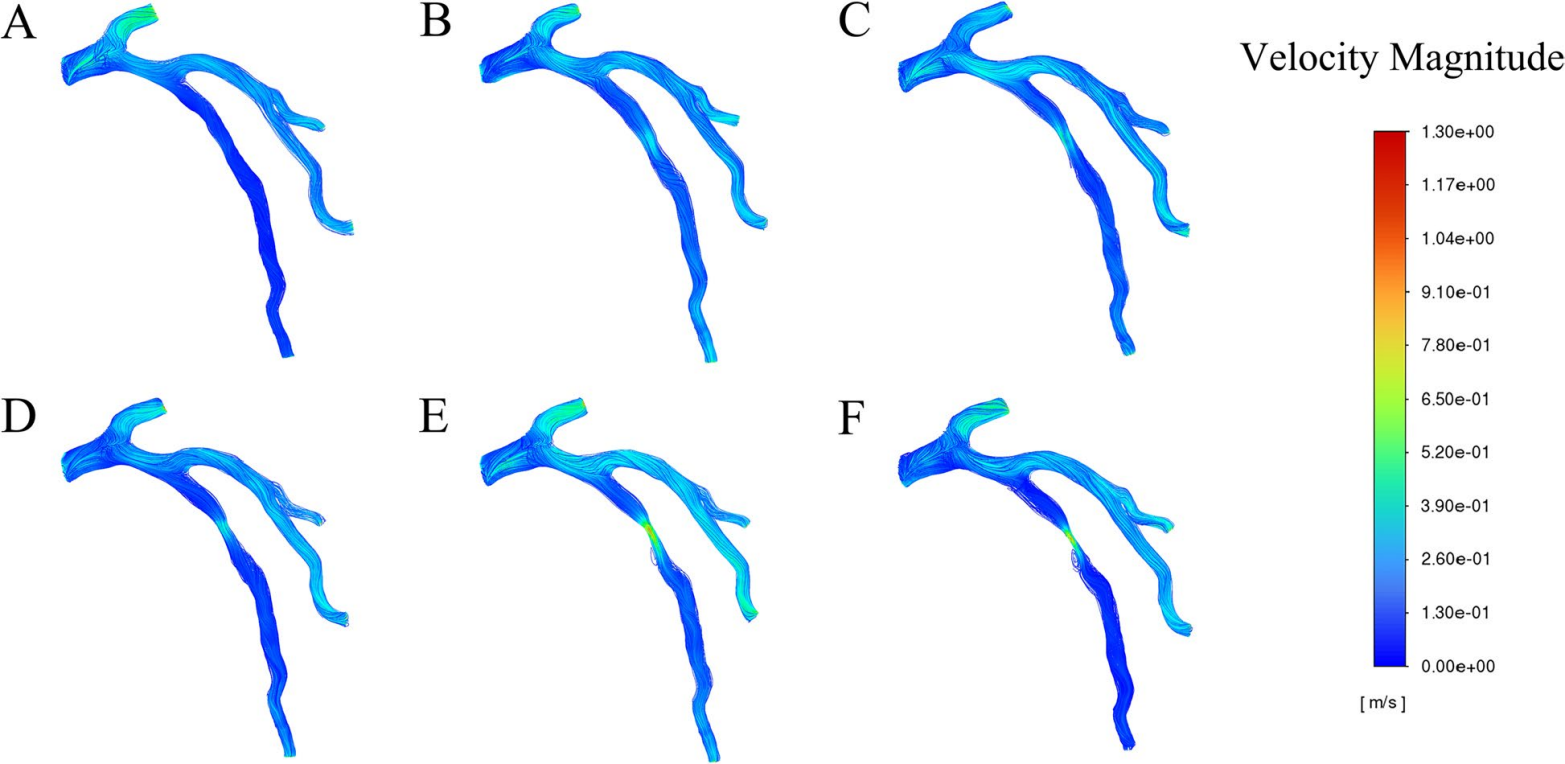
Blood flow velocity distribution in the LAD at the peak systole in a cardiac cycle. **A**. 30% stenosis, **B**. 40% stenosis, **C**. 50% stenosis, **D**. 60% stenosis, **E**. 70% stenosis, **F**. 80% stenosis

**Fig. 9:**
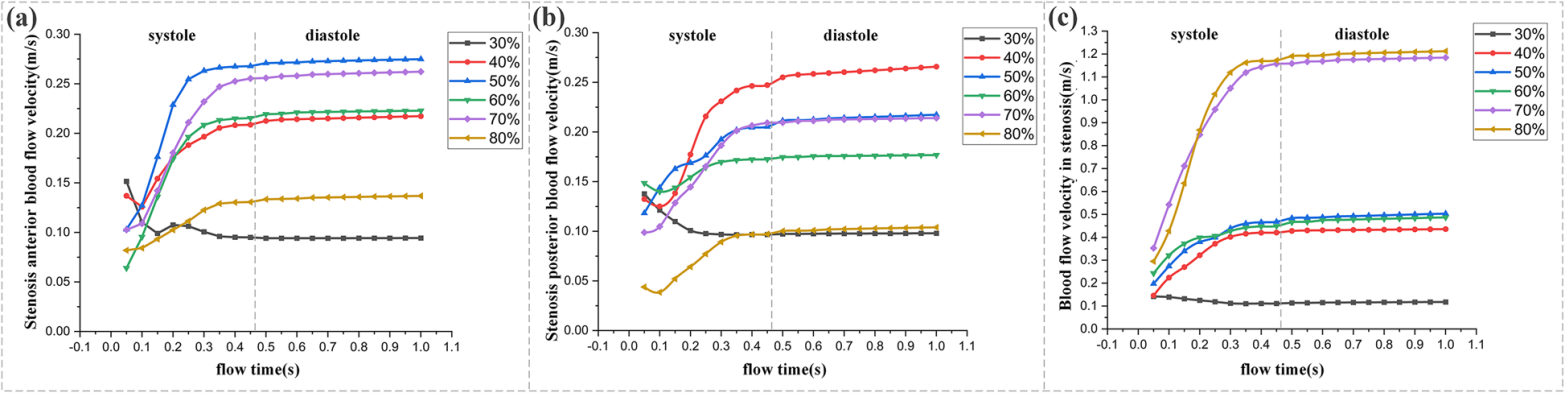
Variation of blood flow velocity distribution of three specific points (**A**, anterior stenosis; **B**, posterior stenosis; **C**, in stenosis (the center) during the cycle

Time-resolved numerical simulation of the coronary stenosis throughout the complete cardiac cycle was obtained, which showed the hemodynamic parameters at the peak systole (t = 0.4 s), whose results stemmed from simulation calculation of six models with diverse severities of stenosis. The hemodynamic parameters, such as pressure, WSS, OSI, RRT and blood flow velocity, were depicted in Figs. 3, 4, 5, 6, 7, 8, 9.

### Pressure

In this study, high pressure was noticed at the blood flow inlet of all models, while at the stenosis and outlet, low pressure was displayed with the obvious gradient downward trend, as shown in Fig. 3. Moreover, with the increase of severity (30%-60% stenosis), the pressure difference between the anterior and posterior portions in the stenotic area ascended slightly with the maximal pressure difference reaching 250pa. Whereas in models (70%, 80% stenosis), the pressure difference rose significantly with the maximum value of 750pa and 1000pa respectively. In addition, the pressure difference remained slight between the center and the posterior portion of the stenotic area in the models with 30%-60% stenosis while the model with 70%-80% stenosis displayed a relatively dramatic pressure gradient with a pressure difference up to 550pa (Fig. 4d).

The real-time changes of pressure throughout the cardiac cycle in different models were tracked for observing the effects of different severities of stenosis on pressure. As demonstrated in Fig. 4a, generally, with the increase of severity (30%-80% stenosis), the pressure in the anterior section of the stenosed area reached the maximum value in the systolic phase, and then it decreased continuously in the early diastolic phase and remained low until the end of the cycle. Furthermore, in Fig. 4b and c, the pressure in the center tended to change almost the same way as that in the posterior portion of the stenosis throughout the complete cardiac cycle in all models, whose pressure reached the peak at the systole, and then it descended at the diastole along with lower values throughout the diastole period. However, the pressure value differed evidently across divergent models: in models with 30%-60% stenosis, the pressure value increased slightly with the aggravation of stenosis, with the maximum blood pressure at the systole fluctuating around 2000pa while in the models with 70%—80% stenosis, the pressure value declined sharply despite the worsening of the stenosis, with the maximum pressure reaching1700pa for the 70% stenosis and 1200pa for the 80% stenosis.

#### WSS

In this study, when the degree of stenosis ranged from 30 to 60%, a slight elevation in wall shear stress (WSS) with the maximum value of 28pa (Fig. 5) was noticed in the center of the stenosed region while relatively lower WSS was found in other portions of the stenosis. However, when stenosis attained 70%, WSS in the center of LAD stenosis ascended sharply, and 80% stenosis even rose more significantly with the peak WSS reaching 80pa. As shown in Fig. 5, there was no significant change in WSS at the outlet and inlet, other bifurcated and curved areas.

#### OSI

According to Fig. 6 (a)-(e), the positions near the branches of the artery or after the stenoses were found to be more prone to higher OSIs, with the peak value reaching 0.49, while the OSI at the stenosis was relatively low. Moreover, as shown by Fig. 5, the distribution of high OSI areas increased with the worsening of stenosis ranging from 30 to 80% degrees, i.e., the higher the narrowing rate, the wider the distribution of high OSI areas.

#### RRT

It could be seen from Fig. 6 that the RRT in front of stenosis was generally high with the peak value reaching 10^7.3^ while it obviously decreased at the stenosis, and rapidly increased after stenosis with the highest value of 10^7^. Moreover, RRT in each model was positively correlated with OSI (Figs. 6, 7), i.e., the distribution of high RRT areas corresponded to that of high OSI areas. Additionally, the peak of OSI approaching 0.5 was often accompanied by a very high RRT value (in principle, when OSI approaches 0.5, RRT approaches infinity). However, as Fig. 7 indicated, unlike the relatively dramatic change of high OSI area, the area of high RRT in the posterior region of stenosis increased slightly along with the aggravation of stenosis.

### Blood flow velocity

As it can be seen in Fig. 8, which showed the blood flow velocity at the peak systole (*t* = 0.4 s), there was a narrow variation range of the blood flow velocity in 30%-40% stenosis models when the blood flow passed through the stenosis. However, in the cases (≥ 50% stenosis), the velocity accelerated rapidly in the stenotic area and reached the maximum, and then it presented a descending trend at the outlet. Especially in the models of 70%-80% stenosis, the velocity in the center of the stenosis was extremely fast, whereas for the outlet, it decreased, leading to blood separation and vortices.

Furthermore, according to the periodic variation diagram of blood flow velocity throughout the cardiac cycle (Fig. 9), generally, the blood flow velocity increased in line with the worsening of stenosis (30%-80%), with higher velocity in the center of the stenosis than that in the anterior and posterior portions. However, as shown in Fig. 9c, the velocity of blood flow varied across different severities of stenosis: in the model of 30% stenosis, the blood flew through the stenotic area with relatively stable velocity with the peak value of 0.12 m/s at the systolic phase while in the cases of 40%-60% stenosis, the blood flow accelerated with the peak velocity of 0.45 m/s. Even in the cases of 70% and 80%, the blood flowed at rather higher velocity with the peak reaching 1.2 m/s in the systolic period.

As shown by Fig. 9a and b, the blood flow velocity in the anterior and posterior portions of the stenosis reached the peak at the systolic phase in each model. However, the velocity of blood flow didn’t increase in line with the aggravation of stenosis. For example, in the cases of 40%-70% stenosis, the peak velocity was over 0.2 m/s in the anterior portion while the peak velocity in 80% stenosis was 0.13 m/s, and the peak velocity in 30% stenosis was 0.1 m/s. Similarly, the blood flow velocity didn’t change in accordance to the worsening of the stenosis in the posterior portion of the stenotic region.

## Discussion

In this study, the LAD models with diverse severities of stenosis were constructed by using CTA data of patients. Based on the models, a numerical simulation of intravascular blood flow was conducted using FFR data as the inlet conditions so as to track the real-time changes of hemodynamic parameters throughout the cardiac cycle. Thus, a preliminary profile of how the hemodynamic parameters varied with the various degrees of stenosis was obtained. Additionally, the authenticity and reliability of the obtained hemodynamics were guaranteed by the fact that FFR data of patients was measured clinically.

As shown by Fig. 3, there was presence of high pressure at the blood flow inlets of models with divergent severities of stenosis whereas low pressure with evident gradient downward trend appeared at the stenosis and the outlet. Furthermore, with the worsening of stenosis, the pressure difference between the anterior and posterior portions in the stenotic area ascended. Especially in models with 70% and 80% stenosis, the pressure difference rose significantly (Fig. 4). Just as previous studies have confirmed, the pressure has the function of driving blood into capillaries and spreading it to the myocardium, and irregular pressure can be sensed sensitively by the endothelial cells which are located on the inner surface of artery wall, and converted into biological signals to maintain the stability of vascular environment [28–33]. When the blood flows through the stenosis, it needs more energy due to the rapid drop in pressure at the stenosis. Moreover, the continued effect of pressure difference caused by stenosis can make the lipid substances, which are carried in blood, more likely to deposit in the low-pressure areas, thus aggravating the stenosis of coronary artery [33, 34].

As for WSS (Fig. 5), it remained slightly higher in the center of the stenotic region than other portions of that region across the models with 30%-60% stenosis. However, in the cases of 70%-80% stenosis, the WSS in the center of the stenosis rose sharply, much higher than that in other portions. These results were in line with previous studies [35]. According to the literature, the alteration of WSS is closely related to the formation of atherosclerotic plaque on the ground that extremely high WSS can injure vascular endothelial cells, which are in contact with blood, affecting the secretion function of endothelial cells, leading to abnormal proliferation of smooth muscle cells, and thus promoting the initiation of vascular atherosclerotic plaques [36–40]. Moreover, the endothelial cells are found to be in a state of injury and exfoliation when WSS exceeds 42pa [41]. In this study, the peak WSS in the center of the stenosis was up to 48pa in 60% stenosis model, while it reached 80pa in the case of 80% stenosis. Such drastic elevation of WSS may be contributed by the high blood velocity in the center of the stenosis, which may increase the permeability of arterial endothelial wall, thus inducing the shedding of endothelial cells and gradual aggregation of platelets, ultimately aggravating the stenosis.

Although our study indicated the appearance of comparatively lower WSS in the areas other than the center of the stenosis across the models with 30%-80% of stenosis, the low WSS may also be harmful, because it could change the direction of monocyte attachment to endothelial cells, most likely to promote the formation of thrombosis. This is why low WSS has been deemed as a recognized marker of atherosclerotic changes and plaque formation, resulting in multiple mechanisms to promote arterial wall remodeling [42, 43].

As shown by Fig. 6, high OSI areas and flow disturbance concentrated in the proximal end of the stenosis, where the vortex appeared. Just as previous studies have shown, high OSI (no exact value but generally > 0.2 or 0.3) could lead to vascular wall damage. And the blood flow disturbance could induce the extensive aggregation of platelet, the damaging of endothelial cells, and the formation of thrombosis and atherosclerosis as well [42, 43]. Moreover, in this study, high OSI areas were evident in all LAD models, even mild stenosis. Accordingly, there may be a risk of thrombotic development and plaque growth. Therefore, although previous studies have mainly focused on the hemodynamic features of more serious stenoses, monitoring abnormal hemodynamic parameters alternation of any extent of stenosis is worthwhile for the benefit of prompt lifestyle or medical intervention since the patients are initially diagnosed, even though the stenoses are classified as mild or moderate [41–45].

Similar to the variation of OSI (Fig. 6), RRT index, which has clearly high values in all cases with different stenoses, tend to be high in the post-stenotic region, where recirculation occurs (Fig. 8). According to the literature, the lipoprotein tends to be higher in the locations with lower WSS, but higher OSI and RRT. Studies have also shown that high OSI and RRT can easily lead to endothelial dysfunction on the ground that WSS vector will change its instantaneous direction during arterial pulsation [45, 46]. In addition, high RRT value will increase the contact time between lipoprotein and blood vessel wall, which will directly affect the interaction between lipoprotein and endothelial surface. Theoretically, the longer the contact time, the more fully the lipoprotein reacts with endothelial cells. Therefore, high RRT will promote the development of atherosclerosis.

As it can be seen in Figs. 8 and 9, blood flow velocity speeds up pursuant to the worsening of stenosis due to the fact that the pressure difference between the anterior and the posterior portions of the stenosis provides basic conditions for the acceleration of blood flow velocity in the center of the stenosis [44–46]. When blood flows at low velocity, it appears in the form of laminar flow with only axial motion. However, when it flows through the stenotic vessel, its velocity accelerates. If the velocity increases to some extent, it will change the laminar flow state gradually [47–51].

Moreover, the results of this study (Figs. 8 and 9) showed that the blood flow velocity, in the cases (≥ 50% stenosis), ascended rapidly in the stenotic area and reached the peak, and then it presented a descending trend at the outlet. Especially, in the cases of 70%-80% stenosis, the blood flowed at extremely fast velocity in the center of the stenosis while it decreased at the outlet, leading to blood separation and vortices. According to the literature, severe stenosis may generate a large area of vortex in the downstream of the stenotic area [47, 51]. With the worsening of stenosis, the local area where vortices occur increases as well as the lingering time, leading to the collision and overlapping of the tangible components in the blood along with the release of kinetic energy, which will contribute to the augmentation of lipid concentration, thus further promoting atherosclerosis and exacerbating stenosis [52–56].

To summarize, the continuous variation of multiple hemodynamic parameters in the coronary blood flow field is supposed to exert a lasting impact on the cardiac blood circulation, vascular pressure load and the degree of vascular endothelial cell injury in a certain period of time [57–60]. As this study has shown, there is no evident variation in pressure, velocity and WSS in the models with less than 50% stenosis whereas there are abrupt and dramatic changes in hemodynamics and their values when the stenosis ascends from 60 to 70%. Furthermore, when the stenosis exceeds 70%, there exists substantial pressure difference, WSS, and blood flow velocity in the center of the stenosis. However, it is worth noting that OSI and RRT appeared with obvious abnormalities even in cases of mild stenosis, indicating the potential further aggravation of atherosclerosis. Moreover, based on the comprehensive analysis of the variation of multiple hemodynamic parameters across different models, we could speculate that there may be a critical point for the variation in the distribution of blood flow field in the coronary artery stenotic region in the cases of 60%—70% stenosis. Once the values of some hemodynamic parameters surpass the critical points, their synthetic effect will cause the rapid worsening of coronary stenosis and speedy development of the disease. Therefore, how to quantitatively evaluate the range of the critical values of hemodynamic parameters in patients, who are in the "gray zone" of stenosis, deserves further studies.

There are certain limitations in this study as well. Comparatively, the sample size is small in this study due to the practicality of managing relatively heavy workload of constructing patient-specific LAD models and simulating the reliable pulsatile flow status by using FFR data which were measured clinically. In the future, if more models with different extent of stenosis are constructed, a more comprehensive and deeper insight into the pathogenesis of the coronary atherosclerosis and stenosis will be obtained. Moreover, although the findings of this study are largely in line with earlier clinical research, more application of the model simulation and its hemodynamic outcomes obtained to clinical research are needed in the future to perform the calibration or validation of the simulation for more practical representation of the hemodynamics.

## Conclusion

To conclude, the time-resolved numerical simulation of the coronary stenosis throughout the complete cardiac cycle based on the patient-specific LAD models, using FFR as initial setting to monitor patient-dependent pressure, could present a dynamic and comprehensive profile of how multi-dimensional hemodynamic parameters vary in accordance with divergent severities of stenosis. Consequently, the findings of this study could serve as an effective reference for the clinicians to have a deeper insight into the pathological mechanism of coronary atherosclerosis and the use of CFD simulation as an additional tool for estimating the complication risks in stenosed coronary vessels.

## Data Availability

All data generated or analyzed during this study are included in this published article.

## Abbreviations

CHD: Coronary heart disease
CFD: Computational fluid dynamics
LAD: Left anterior descending
FFR: Fractional flow reserve
3D: Three-dimensional
WSS: Wall shear stress
OSI: Oscillating shear index
RRT: Relative residence time
